# The Utilization of Acetaminophen for Managing PGE1-Induced Fever in Neonates with Critical Congenital Heart Disease

**DOI:** 10.3390/children11121547

**Published:** 2024-12-20

**Authors:** Başak Kaya, Hasan Akduman, Dilek Dilli, Özkan Kaya, Rumeysa Çitli, Ayşegül Zenciroğlu

**Affiliations:** 1 Department of Neonatology, SBU. Dr. Sami Ulus Maternity and Child Research and Training Hospital, Babur St., Number: 36, Altındag 06080, Turkey; akduman2004@yahoo.com.tr (H.A.); dilek.dilli@sbu.edu.tr (D.D.); rumeysa-1985@hotmail.com (R.Ç.); aysegul.zenciroglu@sbu.edu.tr (A.Z.); 2 Department of Pediatric Cardiology, SBU. Dr. Sami Ulus Maternity and Child Research and Training Hospital, Babur St., Number: 36, Altındag 06080, Turkey; drozkankaya@gmail.com

**Keywords:** neonate, paracetamol, patent ductus arteriosus, prostaglandin fever

## Abstract

Introduction: We aimed to retrospectively evaluate the use of acetaminophen, which may be a risk factor for the ductal canal, in the treatment of fever due to prostaglandin E1 (PGE1) infusion in newborns with critical congenital heart disease (CCHD). Methods: The study included newborns who were followed-up in our neonatal intensive care unit with the diagnosis of critical congenital heart disease, developed fever due to PGE1 infusion and had acetaminophen administered for antipyretic treatment. The patent ductus arteriosus diameters of the patients were evaluated by echocardiographic imaging before intravenous acetaminophen treatment and at the end of the day of acetaminophen treatment. Results: PGE1 fever was observed in 10 (6%) of 156 infants with ductus-dependent congenital heart disease. Intravenous acetaminophen treatment administered as an antipyretic in these infants did not cause the narrowing or closure of the ductal canal diameter, which would lead to clinical decompensation if it was closed, and the patients remained hemodynamically stable until surgery. Conclusions: It can be considered that controlled and rapidly administered intravenous acetaminophen therapy in the management of fever in neonates with congenital heart disease who develop fever as a side effect of high-dose intravenous PGE1 infusion therapy (≥0.3 mcg/kg/min) may prevent hemodynamic decompensation in these critically ill infants, and as a secondary outcome, it can be speculated that avoiding rapid daily increases in PGE1 maintenance infusion doses may be necessary to limit the number of paracetamol administrations in these infants.

## 1. Introduction

Critical congenital heart diseases are severe congenital heart defects that require intervention or surgery in the first few months of life. Although fetal anomaly screening detects approximately 50% to 60% of CHDs, CHD remains a significant cause of mortality and morbidity in neonates [1,2,3]. In critical congenital heart disease, systemic or pulmonary blood flow is dependent on the patency of the ductus arteriosus [4]. In infants with suspected ductus-dependent CHD, the most important intervention to reduce mortality and maintain hemodynamic stability is to maintain ductal patency with PGE1 infusion [5,6,7]. Although PGE1 infusion is a life-saving treatment, unfortunately, short-term (<120 h) and long-term (>120 h) side effects can be observed. PGE1 is among the drugs that are reported to cause frequent side effects in infants with CCHD, and PGE1 side effects are determined according to clinical findings [8]. Short-term side effects are usually dose-related and usually occur within the first 48 h. Short-term side effects frequently include apnea and fever [6]. These side effects are among the factors that cause hemodynamic stress in infants with CCHD (fever, intubation, hypoxia, etc.). Infants in this critical population have structurally altered cardiac anatomy and are prone to conduction system abnormalities secondary to this structural anomaly. The presence of tachycardia associated with hemodynamic stress factors can further worsen the mismatch between oxygen delivery and oxygen consumption, putting infants in a vicious cycle in terms of arrhythmia [9]. This impaired myocardial perfusion and filling of the coronary arteries during the diastolic phase of the heart cycle lead to the triggering of arrhythmias in infants with CCHD [10,11]. Therefore, we know that fever management is of vital importance to break the vicious cycle in these critically ill infants. However, we could not find sufficient data in the literature on the management of PGE1 fever developing in infants with CCHD. It has been suggested that mechanical cooling (e.g., warm water) that can be applied to manage PGE1-related fever in infants with CCHD may generally cause shivering, restlessness, and agitation, further disrupting hemodynamic balance [12]. Another option for fever management is PGE1 synthesis inhibitors, and drugs (such as ibuprofen and acetaminophen) are known to be used in the treatment of ductal closure in premature infants [13,14]. However, we could not find any data in the literature on the use of PGE1 synthesis inhibitors in infants with ductal channel-dependent CHD who receive PGE1 treatment. There is no standard procedure for fever management in these infants receiving PGE1 infusion. In our intensive care unit, we had to administer acetaminophen to a few infants with ductal channel-dependent CCHD who developed fever and arrhythmia secondary to high-dose PGE1-induced fever, and we did not detect any significant change in the patent ductus arteriosus diameter, which is critical for these infants, when we followed it with echocardiographic imaging. Therefore, in order to contribute to the literature, we planned to retrospectively collect and present the infants with rare PGE1 fever and our approach to fever management.

## 2. Materials and Methods

Our study is a retrospective and our hospital ethics committee approval was received (AEŞH-BADEK-2024-060). Among the infants diagnosed with CHD in our neonatal intensive care unit between 2015 and 2022, those with ductal channel-dependency who received PGE1 infusion support were included in the study. The clinical courses of these infants were examined; the study group consisted of infants who developed fever secondary to PGE1 use and who were treated with acetaminophen in fever management. We defined fever as >37.5 °C (axillary) [15,16]. Sinus tachycardia due to fever was diagnosed with a pulse of ≥160 beats/minute and the presence of p waves on electrocardiography, while supraventricular tachycardia was diagnosed with a pulse of ≥180 beats/minute and the absence of p waves on electrocardiography. Babies who were considered to have clinical or proven sepsis and who were started on antibiotic treatment were excluded from the study by examining the infection parameters (white blood cell count in complete blood count, C-reactive protein, interleukin-6, blood, urine and cerebrospinal fluid culture examinations) and clinical findings regarding the babies from the registration system of the Dr. Sami Ulus Gynecology, Obstetrics and Child Health and Diseases Education and Research Hospital for fever etiology, and from nurse observations. After these exclusion criteria, babies who developed fever while receiving PGE1 infusion were included in the study. Demographic and clinical characteristics of the babies included in the study (gestational age, birth weight, mode of delivery, gender, time of CHD diagnosis, time of PGE1 onset, initial dose/maximum dose/cumulative dose of PGE1, duration of PGE1 treatment, day of onset of PGE1-related fever, number of days of PGE1 fever, number of acetaminophen administrations, AST, ALT, INR, aPTT values for the risk of hepatotoxicity after acetaminophen) were obtained retrospectively from patient records. All data were recorded in patient case report forms. The PGE1 infusion doses received by the infants were evaluated in two groups as low-dose (<0.03 mcg/kg/min) and high-dose (≥0.03 mcg/kg/min). In addition, the ductus arteriosus diameters obtained from the echocardiography reports of the period when the infants with CHD and PGE1 fever were admitted to the intensive care unit and received maintenance PGE1 infusion were recorded in the patient data. The dose and duration of PGE1 infusion administered to the infants with CHD in our clinic were determined by the same pediatric cardiologist who performed bedside echocardiography on the infants. Ductal diameter was evaluated by bedside echocardiography on the patient’s first admission to the intensive care unit. Babies thought to have PGE1-related fever were given intravenous acetaminophen at a dose of 10 mg/kg/dose. Babies without persistent fever were given two doses/day, and babies with persistent fever were given intravenous acetaminophen at a dose of 10 mg/kg/dose at a rate of 6 h, repeated for a maximum of 4 doses per day. The maximum recommended dose of paracetamol for infants with persistent fever was 12. In addition, echocardiographic evaluation and ductal diameter measurements were repeated by the same pediatric cardiologist at the end of each acetaminophen-administered day for infants who received acetaminophen. Ductal diameter dimensions were recorded on the case forms before and after acetaminophen treatment. Ductal diameter dimensions were evaluated together with clinical findings before and after acetaminophen treatment.

## 3. Statistics

Descriptive statistics (mean ± standard deviation, percentage) were performed to evaluate the demographic and clinical data of the patients. The statistical program SPSS version 26 was used for all analyses. The normality of the data distribution was evaluated with the Shapiro–Wilk test (*n* < 10). Dependent groups *t*-test statistical analysis was used on the echocardiographically evaluated ductal channel size measurements of infants before and after acetaminophen. *p* < 0.05 was considered statistically significant.

## 4. Results

**Figure 1:**
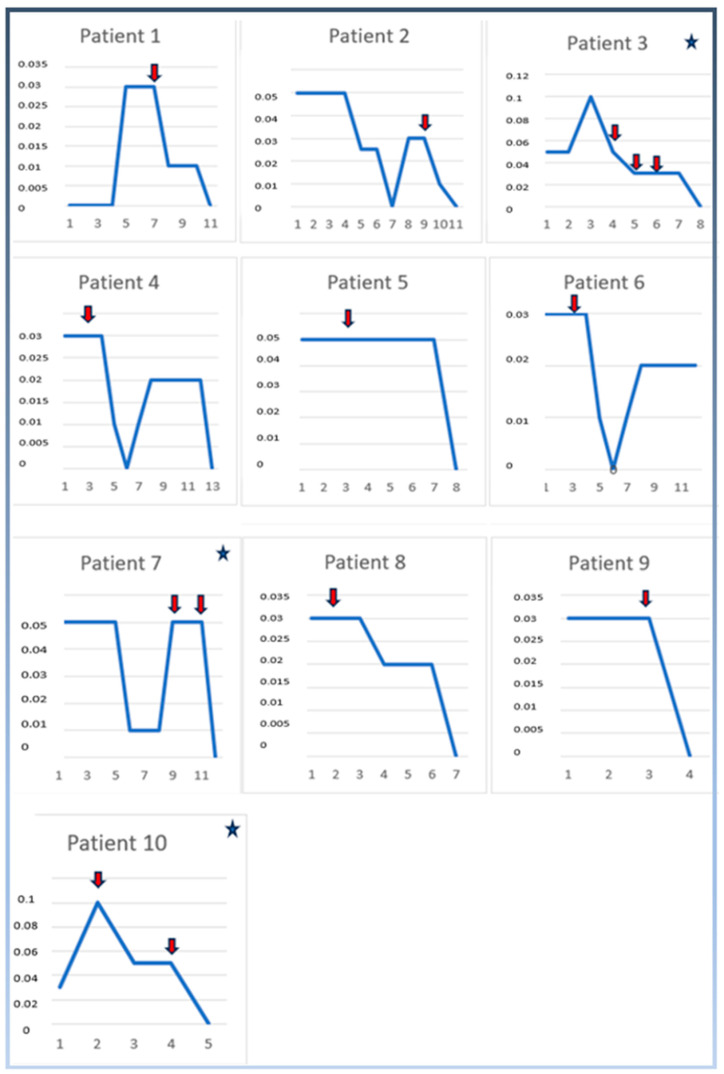
The prostaglandin E1 infusion doses and time to onset of PGE1-related fever. The X-axis represents the days of PGE1 treatment, and the Y-axis represents PGE1 dose (mcg/kg/min). The red arrows indicate when the fever started (how many days after starting PGE1 treatment), and the recurring red arrows indicate on which days the fever recurred. The asterisk for patients 3, 7 and 10 indicates that the fever was persistent.

In our hospital’s neonatal intensive care unit, a total of 156 infants with a diagnosis of CCHD were given PGE1 infusion support between 2015 and 2022. Fever associated with PGE1 infusion was detected in 10 cases out of 156 infants. The demographic characteristics and CCHD diagnoses of the infants with PGE1 fever are presented in Table 1 and Table 2, respectively. In our study, eight of the infants who received high-dose PGE1 and had PGE1 fever were diagnosed with CCHD associated with pulmonary obstruction, one with CCHD associated with systemic obstruction and one with inadequate mixing (TGA) CCHD. Maintenance PGE1 infusion was started at a dose of ≥0.03 mcg/kg/min in 6 of the 10 infants, and 4 at a dose of 0.05 mcg/kg/min. In infants with fever associated with PGE1 infusion, maintenance doses of PGE1 ≥ 0.03 mcg/kg/min (0.03–0.1 mcg/kg/min) were initiated and the mean cumulative PGE1 infusion dose was 0.377 ± 0.170 mg/kg (range: 0.12–0.61). The mean duration of PGE1 infusion support was 7.9 ± 4.3 days (range: 3–16 days). Seven of the ten patients included in the study (patients 1, 2, 4, 5, 6, 8, 9) developed fever within the first 72 h after the initial maintenance dose of PGE1 treatment (patients 1, 4, 5, 6, 8, 9) or within the first 72 h after PGE1 treatment was stopped and restarted (patient 2); these infants were administered 10 mg/kg/dose acetaminophen intravenously twice a day, a total of two doses for a single day, and their fever did not recur during the follow-up. The other three patients (patients 3, 7, 10) developed PGE1 fever within the first 72 h after the PGE1 infusion dose was increased. The fevers of these three infants who developed fever after the PGE1 dose was increased were refractory. Two of these infants (patients 7, 10) received a maximum of four doses per day, a total of eight doses for two days; one of these infants (patient 3) was administered a maximum of four doses per day, a total of twelve doses of 10 mg/kg/dose acetaminophen intravenously for three days, and her fever did not recur during follow-up. Hepatotoxicity was not detected in any infant. The relationship between PGE1 infusion doses and PGE1 fever is shown in Figure 1. Sinus tachycardia observed i the observations of the babies during the febrile period regressed within two hours with acetaminophen treatment. Only one of our patients (DORV, pulmonary atresia diagnosis) had persistent SVT (supraventricular tachycardia) attacks (four attacks) during the PGE1-related fever period; SVT attacks recurred despite adenosine and amiodarone treatments, and the arrhythmia was brought under control by applying synchronized cardioversion (2 joules/kg), adjusting the drug doses, and reducing the body temperature to the normal range with intravenous acetaminophen treatment. In the echocardiographic evaluation performed before and after acetaminophen in the babies whose fever regressed after acetaminophen treatment, no statistically significant difference was found in the channel diameters (*p* > 0.05) (Table 2).

## 5. Discussion

In this study, we observed that fever and fever-related tachycardia developing due to PGE1 infusion in infants with ductus-dependent CCHD resolved after acetaminophen treatment without affecting the diameters of the ductus arteriosus (PDA). In cases of suspected CCHD, it is of vital importance to start PGE1 infusion at an appropriate maintenance dose, and higher doses may be required in some CCHD subtypes. It is well known that high-dose PGE1 administration can cause side effects; apnea and fever are the most common of these side effects [17]. In the study conducted by Alghanem et al., fever was reported in 40% of 435 newborns who received PGE1 treatment, and sepsis was not excluded as an etiology of fever in these patients [18]. Since patients with fever due to sepsis were excluded in our study, we determined the rate of PGE1-related fever as 6.4%. In terms of reducing PGE1 side effects such as apnea and fever, Haughey et al. emphasized that PGE1 infusion doses should be routinely standardized at 0.01 mcg/kg/min [17]. However, PGE1 infusion doses cannot be standardized in all CCHD cases, and Vari et al. found that 17% of infants with ductal channel-dependent CCHD required increased PGE1 and especially those with pulmonary obstruction required higher doses of PGE1 infusion than those with systemic obstruction [19]. The results of our study support the results of the study conducted by Vari et al., and most infants (80%) who received high doses of PGE1 had CCHDs associated with pulmonary obstruction. Even in healthy newborns, conditions such as fever and tachycardia during the transition period may accelerate metabolic activity due to myocardial immaturity and cause circulatory problems, while in newborns with CCHD, these side effects may lead to hemodynamic decompensation (coronary insufficiency, increased catabolic activity) more rapidly, requiring urgent interventions. Therefore, it is important to make a quick decision in case of fever in infants with CCHD. In this context, acetaminophen, which has both antipyretic and analgesic properties, is recommended as a single compound in the treatment of fever in newborns [16,20,21,22]. Acetaminophen can cause side effects such as hepatotoxicity and neurodevelopmental disorders. In studies conducted on infants < 29 and <32 weeks of gestation, no significant difference was found between the groups receiving and not receiving acetaminophen in terms of neurodevelopment [23,24].

Although the risk of hepatotoxicity due to excessive paracetamol exposure is well known in children and adults, hepatotoxicity is rare in the fetal [25,26,27] and neonatal periods [28,29,30]. This situation is due to intracellular enzyme deficiencies and deficiencies in the antioxidant mechanisms of the liver. In the liver, 80–90% of acetaminophen is converted to non-toxic metabolites, and the remaining small percentage is converted to the toxic metabolite N-acetyl-p-benzoquinone imine (NAPQI) [31,32,33,34]. This conversion to toxic metabolites occurs through CYP2E1 expression in pericentral hepatocytes. Low hepatic expression of CYP2E1 in fetuses and neonates constitutes a protective mechanism against liver damage [35,36,37,38,39]. Preclinical and clinical studies on this subject have proven that neonates are resistant to paracetamol-induced liver damage [19,40,41]. No hepatotoxicity was observed in the infants in our study. In our study, refractory arrhythmia triggered simultaneously with PGE1 fever was observed in one case and the arrhythmia was controlled with dual antiarrhythmic therapy, cardioversion and antipyretics. In a study evaluating the thermodynamic effects of intravenous acetaminophen in 99 newborns, it was observed that the mean temperature decrease in the first 2 h after acetaminophen administration in febrile (>37.8 °C) newborns was 0.8 °C [31]. Similar to this study, we observed from nurse observations that the fevers of all our patients responded to acetaminophen treatment and decreased within 1–2 h. However, we had to give repeated doses of acetaminophen to 30% of our patients to manage persistent fever. The cumulative doses of PGE1 administered to these patients with persistent fever were similar. Therefore, we thought that the persistent fever in our patients may be due to the sudden and rapid increase in PGE1 doses rather than the cumulative dose of PGE1. There is no study on this subject in the literature. The rate of increase in the maintenance dose of PGE1 in patients with persistent fever was calculated for those who increased the maintenance dose more than twice in one day. With this study, we can speculate that increasing the PGE1 dose two-fold or more in one day while receiving high-dose PGE1 infusion (≥0.03 mcg/kg/min) may cause persistent fever and increase acetaminophen exposure. In our study, we observed that intravenous acetaminophen treatment given to reduce fever due to PGE1 infusion in infants with CHD did not cause a statistically significant change in PDA diameter. We hypothesize that the persistent patency of PDAs in these infants may be due to the administration of high-dose PGE1 infusions (≥0.03 mcg/kg/min). Based on these findings, we consider that acetaminophen may be administered to infants suffering from CHD with pulmonary obstruction who receive high-dose PGE1 infusions, considering its benefits and harms. A single published case report documented that acetaminophen given as an analgesic for 10 days to an infant with CHD receiving low-dose PGE1 infusions (0.01 and 0.02 mcg/kg/min) resulted in PDA closure. However, this effect was reversed when acetaminophen was discontinued and the PGE1 dose was increased to ≥0.03 mcg/kg/min [42]. In Ryder’s study, the PGE1 dose applied when the PDA was closed was low, and the observation that this negative effect decreased when the dose was increased to a higher dose supports the fact that acetaminophen in our study did not cause ductal effects. In our study, we observed that 12 doses of intravenous paracetamol given for a maximum of 3 days to reduce fever, a common side effect of high-dose PGE1 infusions, did not cause a decrease in PDA diameter in these infants. Another factor we comment on is that the duration of paracetamol treatment for fever in our study was more limited than the duration of application for analgesia in the case report presented by Ryder et al., and so, therefore, were the cumulative doses of PGE1. Since 80% of our patients were infants with right-sided obstructive CCHD associated with PaO2 deficiency, there may be cases where higher-dose PGE1 maintenance infusions may be needed in the initial stage than in this patient group, or where the PGE1 infusion may be rapidly increased to provide oxygenation urgently. Therefore, PGE1 fever side effects may be seen more frequently in infants with right-sided obstructive CCHD.

The limitations of our study are its retrospective nature and the small number of cases. On the other hand, a notable strength of our study is that it is the first study to investigate the management of fever symptoms associated with PGE1 infusion in neonates with PDA-dependent CCHD. This pioneering effort addresses the adverse hemodynamic effects of fever in infants with CCHD, and we suggest that acetaminophen can be used as an antipyretic without any adverse effects on PDA diameter in infants receiving high-dose PGE1.

## Figures and Tables

**Table 1:** Characteristics of infants with CCHD and PGE1-induced fever (*n*: 10).

Maternal age (yrs), mean ± SD	31.6 ± 6.71
Gestational age (weeks), mean ± SD	37.44 ± 1.79
Birth weight (grams), mean ± SD	3187 ± 615.5
Mode of delivery	
Cesarean section, (%)	6 (60)
Gender	
Female, (%)	8 (80)
Prenatal diagnosis of CHD, (%)	2 (20)
Consanguinity, (%)	6 (60)
Referred from outer centers, (%)	8 (80)

**Table 2:** Ductal measurements of study patients before and after acetaminophen treatment.

	Echocardiographic Evaluation	
CCHD Types	PDA Diameter Before Acetaminophen Treatment	PDA Diameter After Acetaminophen Treatment
1. Fallot Tetralogy	large PDA (5 mm)	large PDA (5 mm)
2. HLHS	large PDA (5 mm)	large PDA (4.7 mm)
3. Pulmonary atresia (*)	large PDA (4.2 mm)	large PDA (4 mm; 4.2 mm; 4.1 mm)
4. Pulmonary atresia (*)	large PDA (4.5 mm)	large PDA (4.4 mm)
5. DORV	large PDA (4.8 mm)	large PDA (4.6 mm)
6. TGA	large PDA (6 mm)	large PDA (4.8 mm; 4.9 mm)
7. Tricuspid atresia type 1 (*)	tortuous PDA (4.1 mm)	tortuous PDA (4.1 mm; 4.2 mm)
8. Ebstein anomaly	large PDA (4.8 mm)	large PDA (4.5 mm)
9. Pulmonary atresia	tortuous large PDA (5.5 mm)	tortuous vertical PDA (5.6 mm)
10. Pulmonary atresia, DORV	tortuous vertical PDA (5.2 mm)	vertical PDA (5.3 mm; 5.0 mm)

HLHS, hypoplastic left heart syndrome; DORV, double outlet right ventricle; PDA, patent ductus arteriosus; TGA, transposition of the great arteries. (*) Infants with persistent fever when the PGE1 infusion dose was increased.

## Data Availability

The original contributions presented in this study are included in the article. Further inquiries can be directed to the corresponding author.
